# Histological type and marker expression of the primary tumour compared with its local recurrence after breast-conserving therapy for ductal carcinoma in situ

**DOI:** 10.1054/bjoc.2000.1618

**Published:** 2001-02

**Authors:** N Bijker, J L Peterse, L Duchateau, E C Robanus-Maandag, C A J Bosch, C Duval, S Pilotti, M J van de Vijver

**Affiliations:** Department of Pathology, The Netherlands Cancer Institute, Plesmanlaan 121, Amsterdam, CX, 1066, NL; EORTC Data Center, Avenue E. Mounier 83, bte 11, Brussels, 1200, Belgium; Division of Experimental Therapy, The Netherlands Cancer Institute, Plesmanlaan 121, Amsterdam, 1066 CX, NL; Department of Pathology, Centre Henri Becquerel, 1 Rue d'Amiens, Rouen, 76038, France; Department of Pathology, Istituto Nazionale dei Tumori, Via Venezian 1, Milan, 20133, Italy

**Keywords:** ductal carcinoma in situ, breast-conserving therapy, local recurrence, histology, immunohistochemistry

## Abstract

We have investigated primary ductal carcinomas in situ (DCIS) of the breast and their local recurrences after breast-conserving therapy (BCT) for histological characteristics and marker expression. Patients who were randomized in the EORTC trial 10853 (wide local excision versus excision plus radiotherapy) and who developed a local recurrence were identified. Histology was reviewed for 116 cases; oestrogen and progesterone receptor status, and HER2/ *neu* and p53 overexpression were assessed for 71 cases. Comparing the primary DCIS and the invasive or non-invasive recurrence, concordant histology was found in 62%, and identical marker expression in 63%. Although 11% of the recurrences developed at a distance from the primary DCIS, nearly all these showed the same histological and immunohistochemical profile. 5 patients developed well-differentiated DCIS or grade I invasive carcinoma after poorly differentiated DCIS. Although these recurrences occurred in the same quadrant as the primary DCIS, they may be considered as second primary tumours. Only 4 patients developed poorly differentiated DCIS or grade III invasive carcinoma after well differentiated DCIS. We conclude that in most cases the primary DCIS and its local recurrence are related histologically or by marker expression, suggesting that local recurrence usually reflects outgrowth of residual DCIS; progression of well differentiated DCIS towards poorly differentiated DCIS or grade III invasive carcinoma is a non-frequent event. © 2001 Cancer Research Campaign http://www.bjcancer.com


*British Journal of Cancer* (2001) **84**(4), 539–544
© 2001 Cancer Research Campaign
doi: 10.1054/ bjoc.2000.1618, available online at http://www.idealibrary.com on IDEAL®

http://www.bjcancer.com

### Histological type and marker expression of the primary tumour compared with its local recurrence after breast-conserving therapy for ductal carcinoma in situ

**N Bijker[1], JL Peterse[1], L Duchateau[2], EC Robanus-Maandag[1,3], CAJ Bosch[1,3], C Duval[4], S Pilotti[5] and MJ van de Vijver[1,3]**

[1]The Netherlands Cancer Institute, Department of Pathology, Plesmanlaan 121, 1066 CX Amsterdam, NL; [2]EORTC Data Center, Avenue E. Mounier 83, bte 11, 1200 Brussels, Belgium; [3]Division of Experimental Therapy, The Netherlands Cancer Institute, Plesmanlaan 121, 1066 CX Amsterdam, NL; [4]Centre Henri Becquerel, Department of Pathology, 1, Rue d'Amiens, 76038 Rouen, France; [5]Istituto Nazionale dei Tumori, Department of Pathology, Via Venezian 1, 20133 Milan, Italy

**Summary** We have investigated primary ductal carcinomas in situ (DCIS) of the breast and their local recurrences after breast-conserving therapy (BCT) for histological characteristics and marker expression. Patients who were randomized in the EORTC trial 10853 (wide local excision versus excision plus radiotherapy) and who developed a local recurrence were identified. Histology was reviewed for 116 cases; oestrogen and progesterone receptor status, and HER2/*neu* and p53 overexpression were assessed for 71 cases. Comparing the primary DCIS and the invasive or non-invasive recurrence, concordant histology was found in 62%, and identical marker expression in 63%. Although 11% of the recurrences developed at a distance from the primary DCIS, nearly all these showed the same histological and immuno-histochemical profile. 5 patients developed well-differentiated DCIS or grade I invasive carcinoma after poorly differentiated DCIS. Although these recurrences occurred in the same quadrant as the primary DCIS, they may be considered as second primary tumours. Only 4 patients developed poorly differentiated DCIS or grade III invasive carcinoma after well differentiated DCIS. We conclude that in most cases the primary DCIS and its local recurrence are related histologically or by marker expression, suggesting that local recurrence usually reflects outgrowth of residual DCIS; progression of well differentiated DCIS towards poorly differentiated DCIS or grade III invasive carcinoma is a non-frequent event. © 2001 Cancer Research Campaign http://www.bjcancer.com

**Keywords**: ductal carcinoma in situ; breast-conserving therapy; local recurrence; histology; immunohistochemistry

Mammographic screening has led to a markedly increased detection of ductal carcinoma in situ (DCIS), with 15–20% of all screen-detected breast cancers being DCIS. Breast-conserving therapy (BCT) is increasingly employed for these lesions. Approximately 40–60% of all patients with DCIS are currently offered conservative treatment (Ernster et al, 1996). BCT carries the risk of recurrent disease, which can be the result of outgrowth of the same disease, or represent a new primary tumour. The localization of the recurrence is mainly used to distinguish between these two events. Outgrowth of the same disease (= residual disease) occurs at or near the site of the primary DCIS, whereas lesions developing in another quadrant may be considered new primary tumours. Morphological comparison of the initial tumour with the recurrence may help to distinguish between residual and new disease. Several studies have shown that there is a significant correlation between the histological type of DCIS component adjacent to an invasive carcinoma and the grade of the invasive breast cancer, with well differentiated DCIS being associated with grade I invasive breast cancer, and poorly differentiated DCIS with grade III invasive carcinoma (Lampejo et al, 1994; Goldstein and Murphy, 1996). Therefore, it is likely that if progression from in situ to invasive carcinoma occurs, well differentiated DCIS gives rise to grade I invasive carcinoma, whereas poorly differentiated DCIS gives rise to grade III invasive breast cancer. Different morphology between the primary and the recurrent tumour may be due to the occurrence of a new neoplasm, or due to loss of differentiation features, further referred to as 'dedifferentiation'. It is a matter of debate whether dedifferentiation is a common phenomenon in breast cancer development. A study focusing on histological progression in invasive breast cancer showed a high rate of similarity between the grade of the primary invasive lesion and the subsequent local, nodal or distant recurrence (Millis et al, 1998), suggesting that dedifferentiation is not very likely to occur in breast cancer. No study has investigated progression of DCIS treated with BCT.

We compared localization and histological type of the primary DCIS with the local recurrence in a series of patients treated with BCT for DCIS in the European Organization for Research and Treatment of Cancer (EORTC) trial 10853 (Julien et al, 2000). To support the histological classification, immunohistochemistry was used to assess the expression of oestrogen receptor (ER), progesterone receptor (PR), p53 and HER2/*neu* (c-ErbB2) proteins in the primary DCIS and the recurrence. Well differentiated DCIS and grade I invasive breast cancer are often immuno-positive for ER and PR, whereas poorly differentiated DCIS and grade III invasive breast cancer frequently overexpress HER2/*neu* and p53 (Bobrow et al, 1994; Zafrani et al, 1994; Leal et al, 1995; Perin et al, 1996; Mack et al, 1997).

The objectives of this study were to obtain insight into the incidence of second primary tumours after BCT for DCIS and to

*Received 29 June 2000*
*Revised 6 November 2000*
*Accepted 8 November 2000*

*Correspondence to:* MJ van de Vijver (e-mail: mvijver@nki.nl)

analyse whether progression and dedifferentiation of DCIS in time occurs.

### MATERIALS AND METHODS

### Patients

Histological slides and tissue blocks were collected of patients who were randomized in the EORTC trial 10853 and who developed a local recurrence. Study design, eligibility criteria, treatment, follow-up procedures and definition of endpoints have been described in detail in a report on the first results of the trial (Julien et al, 2000). In this study, 1010 patients had been randomized; at the time of the collection of the material for the current study, 145 recurrences had occurred, half of which were invasive breast cancers. Information about the localization of primary DCIS and local recurrence was obtained from the EORTC Data Center.

### Histology

Slides were collected of 120 of the 145 local recurrences (83%) and were reviewed by one of the authors (JLP), without knowledge of the histological type of the primary lesion. Since the corresponding primary lesion of 116 of the 120 cases had been reviewed previously by the same pathologist as part of a central pathology review for 863 of the randomized cases, histology of the primary lesion and recurrence of these cases could be compared.

DCIS was classified according to the classification described by Holland et al (1994), based on cytonuclear morphology and on architectural patterns, subdividing the lesion into well, intermediately, and poorly differentiated DCIS. Invasive recurrences were classified according to the standard WHO criteria (World Health Organisation, 1981), and graded according to the Bloom and Richardson criteria, modified by Elston and Ellis (1991).

### Immunohistochemistry

For 71 patients the blocks of both the primary DCIS and the recurrence could be collected. Immunohistochemistry was performed on 4 μm-thick sections of formalin-fixed, paraffin-embedded tissues. The sections were mounted on Apes-coated slides and dried. After the sections were dewaxed and endogeneous peroxidase was blocked using 3% hydrogenperoxidase/methanol for 20 minutes, slides were washed in running demi water for 5 minutes. Sections were preincubated for 30 minutes at room temperature (RT) in 1% BSA/PBS (ER, PR) or 5% NGS/PBS (HER2/*neu*, p53). For immunostaining of ER, PR and p53, antigen retrieval was done by boiling for 15 minutes in 10 mM citrate buffer (pH6) in a microwave oven. ER staining was performed using the monoclonal antibody ER1D5, with a dilution of 1:500 (Immunotech, Marseille, France). PR staining was performed using the polyclonal antibody rabbit anti-human PR at a dilution of 1:800 (DAKO, Glostrup, Denmark). HER2/*neu* staining was performed using the monoclonal antibody 3B5, at a dilution of 1:10 000 (Van de Vijver et al, 1988). p53 protein staining was performed using the monoclonal antibody DO-7, at a dilution of 1:8000 (DAKO). Primary antibodies were diluted in 1% BSA/PBS. The sections were incubated with the primary antibody overnight at 4˚C. After washing in PBS, the slides were incubated with biotinylated goat anti-mouse or -rat immunoglobulins (DAKO), diluted to 1:500 in 10% NHS/BSA/PBS, and subsequently with peroxidase-conjugated streptavidin (StreptABComplex/HRP, DAKO), diluted to 1:200 in 0.25% NGS/BSA/PBS, both for 30 minutes at RT. Peroxidase activity was detected by incubation for 5 minutes at RT with 3-3′-diamino-benzidine tetrahydrochloride (DAB), producing a brown staining reaction. Sections were counterstained with haematoxylin. Stainings of the primary lesion and the recurrence were done in pairs in one run, to avoid differences due to technical artefacts.

The immunohistochemical staining was assessed by one pathologist (MvdV), in independent sessions for the primary lesions and recurrences. In those recurrences where both a DCIS and an invasive component were present, these were scored separately.

ER and PR status and HER2/*neu* overexpression were analysed as negative versus any positive staining. For p53 an estimate of the percentage of positive tumour cell nuclei was given using a 6-point scale (0 = 0%, 1 = < 10%, 2 = 10–25%, 3 = 25–50%, 4 = 50–75%, 5 = 75–100%). The mean staining intensity was determined using a 4-point scale (0 = none, 1 = weak, 2 = moderate, 3 = strong). The staining score was calculated as the sum of the mean staining intensity and the percentage of positive tumour cell nuclei (range 0–8). A score of 0 to 4 was determined p53-negative and a score of equal to or greater than 5 was determined p53-positive.

Positive and negative controls were included in every run. Tumours with known expression of ER, PR, p53 and/or HER2/*neu* were used as positive controls. Normal breast tissue (present in most cases) served as an internal control for ER and PR stainings.

In some cases the amount of tumour in the block was insufficient for staining of all 4 markers, therefore the results of 4 pairs for ER, 5 for PR, 4 for HER2/*neu*, and 2 for p53 immunostaining are not included.

### Statistical analysis

Spearman's ranked correlation was used to evaluate the relation between the histological type of the primary DCIS and the recurrence. The strength of correlation was determined by the weighted kappa statistic. The simple kappa statistic was used for the correlation of the marker expression of the primary and recurrent lesion, since here there are only two values for each marker. Values of kappa range from 0 for chance agreement only to +1 for perfect correlation. A kappa value of 0 to 0.20 indicates a weak correlation, 0.21 to 0.40 fair, 0.41 to 0.60 moderate, 0.61 to 0.80 good, and 0.81 to 1.00 a very good correlation (Altman, 1997).

### RESULTS

61 of the 116 recurrences were DCIS (53%) and 55 were invasive (47%). 44 patients (38%) had been treated with excision followed by radiotherapy (25 DCIS, 19 invasive recurrences), and 72 patients by excision only (62%) (36 DCIS, and 36 invasive recurrences). The median time to recurrence was 36 months (range, one to 146 months); the median time to a DCIS recurrence was 26 months and to an invasive recurrence 41 months. 13 of the 116 recurrences (11%) occurred in a different quadrant to the primary DCIS; all other recurrences were located at or near the site of the original excision.

### Histology

The histological type of the primary DCIS was well differentiated in 26 (22%), intermediately differentiated in 33 (29%) and poorly

© 2001 Cancer Research Campaign differentiated in 57 (49%) cases. Of the 55 invasive recurrences, 49 were invasive ductal, 2 invasive lobular, one mixed ductulo-lobular and 3 mucinous carcinomas.

41 of the 55 invasive recurrences had an associated DCIS component. The mean diameter of the invasive carcinomas was 12 mm (range, 1 to 30 mm); the size of the invasive recurrence was not significantly different for those recurrences whose primary DCIS was well, intermediately or poorly differentiated.

There was a moderate correlation between the histological type of the primary and the recurrent DCIS (kappa = 0.56, Table 1A), the correlation between the primary DCIS and the invasive recurrence was weaker (kappa = 0.33, Table 1B).

Of 61 DCIS recurrences, 43 (70%) were of identical histological type compared with the primary DCIS. The grade of the invasive recurrence matched with the histological type of the primary DCIS in 29 cases (53%). Of note, one poorly differentiated DCIS, and 3 grade III invasive carcinomas developed after a well differentiated primary DCIS. Furthermore, one well differentiated DCIS, and 4 grade I invasive carcinomas developed after a poorly differentiated DCIS. All these recurrences occurred in the same quadrant as the primary lesion.

Two of the 17 invasive recurrences (12%) after well differentiated DCIS were axillary lymph node positive, 5 of the 14 (36%) after intermediately differentiated DCIS, and 11 of the 24 (46%) after poorly differentiated DCIS. None of the 3 grade III invasive recurrences after well-differentiated DCIS were lymph node positive.

**Table 1A** Histological type primary DCIS related to histological type recurrent DCIS

| | | DCIS recurrence | | | |
|---|---|---|---|---|---|
| | | Well | Intermediate | Poor | Total |
| Primary DCIS | Well | 5 | 3 | 1 | 9 |
| | Intermediate | 3 | 8 | 8 | 19 |
| | Poor | 1 | 2 | 30 | 33 |
| | Total | 9 | 13 | 39 | 61 |

Spearman Correlation 0.65. Weighted Kappa 0.56 (95% CI 0.38–0.73).

**Table 1B** Histological type primary DCIS related to grade invasive recurrence

| | | Grade invasive recurrence | | | |
|---|---|---|---|---|---|
| | | I | II | III | Total |
| Primary DCIS | Well | 8 | 6 | 3 | 17 |
| | Intermediate | 3 | 7 | 4 | 14 |
| | Poor | 4 | 6 | 14 | 24 |
| | Total | 15 | 19 | 21 | 55 |

Spearman Correlation 0.38. Weighted Kappa 0.33 (95% CI 0.13–0.54).

When DCIS and invasive recurrences are taken together, 72 (62%) had the same histological type/grade as the primary DCIS. In the invasive recurrences, the grade of the infiltrating tumour was compared with its associated DCIS component: none of the grade III invasive carcinomas had an associated well differentiated DCIS, and vice versa.

### Immunohistochemistry

40 of the 71 recurrences on which immunohistochemical staining was performed were DCIS (56%) and 31 were invasive (44%). 27 patients (38%) had been treated with excision and radiotherapy, and 44 (62%) by excision only. Table 2 shows the association between the histological type and the marker expression of the primary DCIS. Well differentiated DCIS was associated with ER and PR immuno-positivity; poorly differentiated DCIS was related with HER2/*neu* and p53 overexpression.

Table 3 shows the marker expression of the primary DCIS related to the recurrence. For ER, HER2/*neu* and p53 there was a good to very good correlation, for PR the correlation was moderate.

In 36 of 57 cases (63%) for which ER, PR, HER2/*neu* and p53 could all be immunostained, the marker expression of the primary DCIS was identical to that of the recurrence.

The cases with a difference in the histological type and/or in the marker expression are listed in Table 4. In only 5 cases (9%) more than one marker differed in its expression.

When ER or PR expression changed from negative to positive, or when HER2/*neu* or p53 expression changed from positive to negative, recurrent lesions can be considered a second primary tumour, which was the case in 22 lesions. In only one case all markers differed (case 31, Table 4). This patient developed well differentiated DCIS after poorly differentiated DCIS, which is therefore likely to be a second primary lesion, even though it occurred in the same quadrant.

9 of 71 recurrences showed dedifferentiation with respect to marker expression, i.e. loss of ER or PR immuno-positivity or gain of HER2/*neu* or p53 overexpression. In only one recurrence (case 39) the histological type of the lesion and the marker expression both changed toward a more dedifferentiated lesion. In the other cases, either the histology between the primary and recurrent lesion differed and the marker expression was identical (15 of 26 cases (58%)), or the marker expression differed and the histology was the same (14 of 25 cases (56%), Table 4).

In 25 of 57 cases (44%) for which histology and marker expression were both completely evaluated, the primary and recurrent lesion had identical histology and marker expression.

Case 15, with a poorly differentiated DCIS recurrence developing after a well differentiated DCIS, had identical marker expression in the two lesions. In case 35, with a grade III invasive carcinoma after a well differentiated DCIS, PR expression changed from negative to positive.

**Table 2** Histological type related to marker expression primary DCIS

| | | ER N positive (%) | PR N positive (%) | HER2/*neu* N positive (%) | p53 N positive (%) |
|---|---|---|---|---|---|
| Primary DCIS | Well | 13/13 (100) | 9/13 (69) | 1/12 (8) | 1/13 (8) |
| | Intermediate | 17/21 (81) | 13/20 (65) | 5/19 (26) | 3/21 (14) |
| | Poor | 14/36 (39) | 10/35 (29) | 24/34 (71) | 10/36 (28) |

© 2001 Cancer Research Campaign

All 5 immunohistochemically analysed lesions that had recurred in another quadrant showed either the same histology or identical marker expression compared with the primary lesion (Table 4).

**Table 3** Marker expression of primary DCIS related to recurrence

| Primary DCIS | | Recurrence Positive | Recurrence Negative | Kappa (95% CI) |
|---|---|---|---|---|
| ER | positive | 40 | 1 | |
| | negative | 2 | 23 | 0.90 (0.80–1.01) |
| PR | positive | 24 | 8 | |
| | negative | 8 | 26 | 0.54 (0.33–0.74) |
| HER2/*neu* | positive | 24 | 7 | |
| | negative | 1 | 33 | 0.75 (0.60–0.91) |
| p53 | positive | 9 | 5 | |
| | negative | 0 | 55 | 0.74 (0.53–0.95) |

CI = confidence interval.

An effect of radiotherapy on the change of histological type or marker expression in the recurrent lesion could not be observed (data not shown).

### DISCUSSION

In this study we have analysed primary DCIS and their recurrences after breast-conserving therapy in patients treated as part of a randomized clinical trial (EORTC 10853). The main goals of this study were to obtain insight into the incidence of second primary tumours and to investigate whether 'dedifferentiation' from well to poorly differentiated cancers occurs.

11% of the recurrences developed in a different quadrant to the primary lesion. Clinically, this suggests that these cases are second primary tumours. However, neither by morphology nor by immunohistochemistry was support found that these recurrences were new primary lesions. Establishing the relation between the

**Table 4** Cases listed in which marker expression and/or histology of primary DCIS was not identical to recurrence

| | Primary DCIS | | | | | Recurrence | | | | | | |
|---|---|---|---|---|---|---|---|---|---|---|---|---|
| Patient | Histological type | ER | PR | HER2 | p53 | Quadrant | Histological type | Grade invasion | ER | PR | HER2 | p53 |
| Histology similar, markers different | | | | | | | | | | | | |
| 1 | well | + | – | – | – | same | well | | + | + | – | – |
| 2 | poor | + | + | – | + | other | poor | | – | – | – | + |
| 3 | poor | – | – | – | – | same | poor | | – | + | – | – |
| 4 | intermediate | + | + | – | + | same | intermediate | | + | – | – | – |
| 5 | poor | + | – | + | + | same | poor | | + | – | – | + |
| 6 | poor | + | + | + | – | same | poor | | + | – | + | – |
| 7* | well | + | – | – | – | same | well | | | + | – | – |
| 8* | poor | + | + | – | – | other | poor | | | | + | |
| 9 | poor | – | – | + | – | same | poor | 3 | – | – | – | – |
| 10 | intermediate | + | + | – | – | same | intermediate | 2 | + | – | – | – |
| 11 | intermediate | + | + | – | – | same | intermediate | 2 | + | – | – | – |
| 12 | poor | + | – | – | – | same | poor | 3 | + | + | – | – |
| 13 | poor | – | – | + | – | same | | 3 | + | – | + | – |
| 14 | intermediate | + | + | + | – | other | | 2 | + | + | – | – |
| Markers similar, histology different | | | | | | | | | | | | |
| 15 | well | + | + | – | – | same | poor | | + | + | – | – |
| 16 | poor | – | – | + | – | same | intermediate | | – | – | + | – |
| 17 | intermediate | + | + | – | – | same | well | | + | + | – | – |
| 18 | intermediate | + | + | – | – | same | well | | + | + | – | – |
| 19 | intermediate | + | + | – | – | same | poor | | + | + | – | – |
| 20* | intermediate | – | – | | + | same | poor | | – | – | | + |
| 21* | intermediate | + | + | – | – | same | poor | | | | – | – |
| 22 | intermediate | + | + | – | – | other | intermediate | 1 | + | + | – | – |
| 23 | well | + | + | – | – | other | intermediate | 1 | + | + | – | – |
| 24 | well | + | + | – | – | same | intermediate | 1 | + | + | – | – |
| 25 | poor | + | + | – | – | same | intermediate | 3 | + | + | – | – |
| 26* | poor | + | | – | – | same | intermediate | 2 | + | – | – | – |
| 27* | poor | – | – | | + | same | intermediate | 3 | – | – | | + |
| 28 | intermediate | + | + | – | – | unknown | | 3 | + | + | – | – |
| 29* | intermediate | + | | – | – | same | | 3 | + | + | – | – |
| Histology and markers different | | | | | | | | | | | | |
| 30 | intermediate | + | – | – | – | same | poor | | + | + | – | – |
| 31 | poor | – | – | + | + | same | well | | + | + | – | – |
| 32 | intermediate | – | – | + | – | same | poor | | – | – | – | – |
| 33 | intermediate | + | + | + | – | same | poor | | + | + | – | – |
| 34* | intermediate | + | + | – | – | unknown | intermediate | 1 | – | – | | – |
| 35* | well | + | – | | – | same | poor | 3 | + | + | – | – |
| 36 | poor | + | + | + | + | same | poor | 2 | + | + | + | – |
| 37 | well | + | + | + | – | same | intermediate | 2 | + | + | – | – |
| 38 | poor | + | – | – | – | same | poor | 2 | + | + | – | – |
| 39 | well | + | + | – | – | same | | 2 | + | – | – | – |
| 40 | well | + | + | – | + | same | | 2 | + | – | – | – |

+ = positive expression; – = negative expression; * = not all markers scored.

*© 2001 Cancer Research Campaign*

recurrence and the primary lesion by clinical comparison of the localization of the two lesions is difficult, especially when there is apparently no anatomical relation between the two. DCIS grows typically unicentric, and spreads along one of the 15–20 branching trees forming the glandular breast tissue. These trees are anatomically ill-defined, and often exceed the borders of a quadrant. This may give rise to recurrences remote from the primary lesion, still developing in the same tree, thus being outgrowth of residual disease.

We observed 5 patients with a well differentiated DCIS recurrence or grade I invasive carcinoma evolving after a poorly differentiated DCIS. Although all these recurrences occurred at the same quadrant as the primary DCIS, it is likely that these cases are second primary tumours based on the different histology. In fact, in one of these cases the marker expression analysis showed that the status of ER and PR turned from negative to positive, and the overexpression of p53 and HER2/*neu* from the primary lesion was lost in the recurrence, which is consistent with the morphological alteration. Alternatively, by incomplete eradication of the rarely observed histologically heterogeneous tumours, the well differentiated component may have been left behind (Goldstein and Murphy, 1996).

Our findings show concordant histology in 62%, and identical marker expression in 63% between the primary DCIS and the invasive or non-invasive recurrence. These results suggest that the majority of recurrences after BCT for DCIS reflect residual disease. When both could be scored, the histology and marker expression in primary DCIS and local recurrence was identical in 44%.

It has not been resolved whether dedifferentiation in breast cancer is a common phenomenon, and thus whether a well differentiated DCIS can recur as a higher grade lesion.

A considerable number of recurrent lesions differed one grade from the primary DCIS, resulting in a higher-grade recurrence (i.e. grade II invasive carcinoma after a well-differentiated DCIS), but also frequently in a lower-grade recurrence (i.e. those with an intermediately differentiated recurrence after a poorly differentiated DCIS). We do not believe such differences to be signs of dedifferentiation or new tumour development, but as an expression of the weakness of the three-tier classification system of DCIS. Inter-observer variability in classifying DCIS is a well-known problem (Sloane et al, 1998). Especially for intermediately differentiated DCIS, low consistency is usually obtained. Inter-observer variation in classification of the extremes occurs less frequently, because these are easier to recognize. When this is taken into account, the observed differences are less evident.

4 patients developed a poorly differentiated DCIS or a grade III invasive carcinoma after a well differentiated DCIS. In these cases progression might have occurred, although in two of these cases the marker expression did not confirm this progression towards a higher grade. In both, the marker expression of the recurrence was consistent with the primary well differentiated DCIS (ER/PR positive, HER2/p53 negative). In the other two cases, the marker expression could not be evaluated. Also, the axillary lymph node status was negative in all 4 recurrences. In only one other recurrence, both the histological type and the marker expression changed towards a higher-grade lesion.

If well-differentiated DCIS frequently progressed to grade III recurrences, this would be a reason for more aggressive treatment for this type of lesion. However, although dedifferentiation can occasionally occur, it was an uncommon phenomenon in our study.

Other techniques investigating the relation between primary and recurrent breast malignancies have been described recently. In a study investigating loss of heterozygosity (LOH) in 3 cases of DCIS and their local DCIS recurrences a high concordance has been demonstrated between the lesions, although genetic progression was shown in all 3 by additional LOH in the recurrent lesion (Lininger et al, 1998). More recently, a high concordance in chromosomal alterations in 17 of 18 cases of initial DCIS and their subsequent DCIS recurrences was shown with comparative genomic hybridization (CGH), indicating a clonal relationship between the two events (Waldman et al, 2000). However, the investigators also found a small, but statistically significant, increase in the number of genetic alterations in the recurrences, suggesting dedifferentiation.

The grade of invasive breast carcinoma has been shown to be an independent prognostic factor for recurrence and survival (Elston and Ellis 1991). Gupta et al (1997) have shown that the histological type of DCIS, in the presence of an invasive tumour, correlates with the clinical outcome of the patient. If the histological type of the primary DCIS correlates with the grade of the subsequent invasive recurrence, the histological type of DCIS is also likely to have prognostic implications, with a higher risk of a more aggressive behaviour of local recurrence after poorly differentiated compared to local recurrence after well differentiated DCIS.

Our results on basis of histology and marker expression contribute to obtain insight into the type of recurrence after BCT for DCIS. Comparison of genetic alterations between the primary tumours and their invasive and non-invasive recurrences will help to further define these lesions in order to offer optimal clinical treatment of the various subtypes of DCIS.

### REFERENCES

Altman DL (1997) *Practical statistics for medical research*, p 406. Chapman & Hall: London

Bobrow LG, Happerfield LC, Gregory WM, Springall RD and Millis RR (1994) The classification of ductal carcinoma in situ and its association with biological markers. *Semin Diagn Pathol* **11**: 199–207

Elston CW and Ellis IO (1991) Pathological prognostic factors in breast cancer. I. The value of histological grade in breast cancer: experience from a large study with long-term follow-up. *Histopathology* **19**: 403–410

Ernster VL, Barclay J, Kerlikowske K, Grady D and Henderson C (1996) Incidence of and treatment for ductal carcinoma in situ of the breast. *JAMA* **275**: 913–918

Goldstein NS and Murphy T (1996) Intraductal carcinoma associated with invasive carcinoma of the breast. A comparison of the two lesions with implications for intraductal carcinoma classification systems. *Am J Clin Pathol* **106**: 312–318

Gupta SK, Douglas-Jones AG, Fenn N, Morgan JM and Mansel RE (1997) The clinical behavior of breast carcinoma is probably determined at the preinvasive stage (ductal carcinoma in situ). *Cancer* **80**: 1740–1745

Holland R, Peterse JL, Millis RR, Eusebi V, Faverly D, Van de Vijver MJ and Zafrani B (1994) Ductal carcinoma in situ: a proposal for a new classification. *Semin Diagn Pathol* **11**: 167–180

Julien JP, Bijker N, Fentiman IS, Peterse JL, Delledonne V, Rouanet P, Avril A, Sylvester R, Mignolet F, Bartelink H and Van Dongen JA (2000) Radiotherapy in breast-conserving treatment for ductal carcinoma in situ: first results of the EORTC randomised phase III trial 10853. EORTC Breast Cancer Cooperative Group and EORTC Radiotherapy Group. *Lancet* **355**: 528–533

Lampejo OT, Barnes DM, Smith P and Millis RR (1994) Evaluation of infiltrating ductal carcinomas with a DCIS component: correlation of the histologic type of the in situ component with grade of the infiltrating component. *Semin Diagn Pathol* **11**: 215–222

Leal CB, Schmitt FC, Bento MJ, Maia NC and Lopes CS (1995) Ductal carcinoma in situ of the breast. Histologic categorization and its relationship to ploidy and immunohistochemical expression of hormone receptors, p53, and c-erbB-2 protein. *Cancer* **75**: 2123–2131

Lininger RA, Fujii H, Man YG, Gabrielson E and Tavassoli FA (1998) Comparison of loss of heterozygosity in primary and recurrent ductal carcinoma in situ of the breast. *Mod Pathol* **11**: 1151–1159

*© 2001 Cancer Research Campaign* 

Mack L, Kerkvliet N, Doig G and O'Malley FP (1997) Relationship of a new histological categorization of ductal carcinoma in situ of the breast with size and the immunohistochemical expression of p53, c-erb B2, bcl-2, and ki-67. *Hum Pathol* **28**: 974–979

Millis RR, Barnes DM, Lampejo OT, Egan MK and Smith P (1998) Tumour grade does not change between primary and recurrent mammary carcinoma. *Eur J Cancer* **34**: 548–553

Perin T, Canzonieri V, Massarut S, Bidoli E, Rossi C, Roncadin M and Carbone A (1996) Immunohistochemical evaluation of multiple biological markers in ductal carcinoma in situ of the breast. *Eur J Cancer* **32A**: 1148–1155

Sloane JP, Amendoeira I, Apostolikas N, Bellocq JP, Bianchi S, Boecker W, Bussolati G, Coleman D, Connolly CE, Dervan P, Eusebi V, De Miguel C, Drijkoningen M, Elston CW, Faverley D, Gad A, Jacquemier J, Lacerda M, Martinez-Penuela J, Munt C, Peterse JL, Rank F, Sylvan M, Tsakraklides V and Zafrani B (1998) Consistency achieved by 23 European pathologists in categorizing ductal carcinoma in situ of the breast using five classifications. European Commission Working Group on Breast Screening Pathology. *Hum Pathol* **29**: 1056–1062

Van de Vijver MJ, Peterse JL, Mooi WJ, Wisman P, Lomans J, Dalesio O and Nusse R (1988) Neu-protein overexpression in breast cancer. Association with comedo-type ductal carcinoma in situ and limited prognostic value in stage II breast cancer. *N Engl J Med* **319**: 1239–1245

Waldman FM, DeVries S, Chew KL, Moore DH 2nd, Kerlikowske K and Ljung BM (2000) Chromosomal alterations in ductal carcinomas in situ and their in situ Recurrences. *J Natl Cancer Inst* **92**: 313–320

World Health Organisation (1981) *Histologic typing of breast tumors*, 2nd ed. WHO: Geneva

Zafrani B, Leroyer A, Fourquet A, Laurent M, Trophilme D, Validire P and Sastre-Garau X (1994) Mammographically-detected ductal in situ carcinoma of the breast analyzed with a new classification. A study of 127 cases: correlation with estrogen and progesterone receptors, p53 and c-erbB-2 proteins, and proliferative activity. *Semin Diagn Pathol* **11**: 208–214

*© 2001 Cancer Research Campaign*
